# Glanzmann's Thrombasthenia Diagnosed following Knee Arthroscopy

**DOI:** 10.1155/2015/127846

**Published:** 2015-04-27

**Authors:** John E. Zvijac, Sharhabil S. Ammus, Fernando Aran, Gary M. Kiebzak

**Affiliations:** ^1^UHZ Sports Medicine Institute, Doctors Hospital, Baptist Health South Florida, 1150 Campo Sano Avenue, Suite 200, Coral Gables, FL 33146, USA; ^2^UM Sylvester Cancer Center at Kendall, 8932 SW 97 Avenue, Suite B12, Miami, FL 33176, USA; ^3^FIU Herbert Wertheim College of Medicine, Miami, FL 33199, USA; ^4^Center for Research and Grants, Doctors Hospital, Baptist Health South Florida, 1150 Campo Sano Avenue, Suite 200, Coral Gables, FL 33146, USA

## Abstract

A 41-year-old man with an unremarkable medical history presented with a painful knee after a sports injury. He was diagnosed with a medial meniscal tear. Symptoms did not abate after 6 months of physical therapy, and he underwent arthroscopic partial medial meniscectomy. A week after beginning physical therapy he experienced a knee effusion, decreased ROM, and inability to flex his quadriceps. His knee was aspirated. Blood tests were ordered and his complete blood count, liver functions tests, and INR/PTT were normal. The patient had recurrent effusions requiring three additional joint aspirations. Ten weeks after the initial surgery, the patient underwent a second arthroscopy, during which a hematoma was removed and a synovectomy performed. The patient continued bleeding from the incisions after portals were sutured, and he was admitted to the hospital. A hematologist was consulted and comprehensive platelet aggregation testing revealed previously undiagnosed Glanzmann's thrombasthenia. The patient began treatment with platelet infusions and desmopressin and progressed to a full recovery. Clinical suspicion for surgical patients with unusual repetitive postoperative bleeding should include previously undetected rare bleeding disorders even in adults.

## 1. Introduction

Minor hemarthrosis (bleeding in the joint space) and pain after arthroscopic knee surgery are not uncommon and are usually self-limiting as healing progresses. Occasionally, various events such as failure of the surgical repair, infection, or new traumatic injury, or unrecognized hypertension, use of anticoagulation medications, or liver disease affecting synthesis of clotting factors may result in more serious and repetitive hemarthrosis. Typically, however, the surgeon does not suspect rare clotting disorders in adults with normal results of blood tests as such inherited or acquired disorders are usually detected early in life. In this report, we describe a case of postoperative hemarthrosis after arthroscopic meniscectomy in an ostensibly healthy adult man who was later diagnosed with Glanzmann's thrombasthenia.

## 2. Case Presentation

A 41-year-old man with an unremarkable medical history presented to the clinic with right knee pain, severity 3/10, piercing and sharp in quality, which occurred after recreational sports activity. He was diagnosed with a medial meniscal tear confirmed by MRI. Six months of physical therapy did not relieve the symptoms. The patient underwent diagnostic arthroscopy, partial medial meniscectomy, chondroplasty of the trochlea, and partial synovectomy without intraoperative complication (bleeding was controlled by tourniquet). He started a home exercise program and formal strengthening therapy began on postoperative day 10. Then, in the absence of any particular traumatic event, he experienced a knee effusion, decreased range of motion, and inability to flex his quadriceps muscle on postoperative day 17.

Upon physical exam in the clinic, mild effusion of the right knee was noted with no ecchymosis, and laxity tests and meniscal exams were negative. Range of motion was 2 degrees of extension and 110 degrees of flexion. Knee aspiration was done with 62 mL of serosanguinous fluid withdrawn. The patient was taking aspirin and told to discontinue its use. He was instructed to stop physical therapy, rest his knee, use ice for 20 minutes 3-4 times per day, and wear a compression wrap until his next office visit in 2 weeks. Subsequent blood test results showed normal complete blood count, liver function tests, and INR/PTT.

Within the next several weeks, the patient had recurrent effusions with related symptoms requiring three additional joint aspirations. Ten weeks after the initial surgery, the patient underwent a second arthroscopy, during which a hematoma was removed and a synovectomy performed. The procedure was routine with no intraoperative complications. However, after the procedure the patient continued bleeding from the sutured incisions and was admitted to the hospital for observation. A hematologist was consulted. The patient denied previous bleeding problems. Platelet aggregation tests revealed decreased platelet aggregation and secretion with arachidonic acid, collagen, adenosine diphosphate (ADP), and thrombin. In contrast, platelet aggregation was normal in the presence of ristocetin. These results were consistent with the diagnosis of Glanzmann's thrombasthenia.

After the definitive diagnosis was made, the patient began treatment with platelet infusions and desmopressin and progressed to a full recovery.

## 3. Discussion

Glanzmann's thrombasthenia, first described in 1918 as a hereditary hemorrhagic thrombasthenia, is a rare autosomal recessive bleeding disorder with an estimated incidence of 1 in 1,000,000 [1–3]. It is characterized by normal platelet count but lack of normal platelet aggregation [3]. The platelet function disorder is caused by qualitative and/or quantitative defects in the platelet glycoprotein IIb/IIIa complex (an integrin coded by the* ITGA2B* and* ITGB3* genes and which by binding fibrinogen and other adhesive proteins joins platelets together in aggregate) [4]. Typical symptoms, which may be constant problems in fully expressed cases, include purpura, epistaxis, gingival hemorrhage, menorrhagia, and prolonged bleeding after minor injuries [1]. Logically, excessive bleeding during or after surgery would be expected. It is not associated with early mortality except in cases where excessive bleeding would be expected as with intracranial hemorrhage or visceral hematoma. Diagnosis occurs at an early age (usually before the age of 5 years in a cohort of 113 patients as discussed by George et al. [1]), especially if a family member has been previously diagnosed. Glanzmann's thrombasthenia is a lifelong condition for which there is no cure. Symptoms may be minimized by avoiding use of aspirin or other nonsteroidal anti-inflammatory medications, such as ibuprofen and naproxen [3].

Laboratory tests are necessary to diagnose Glanzmann's thrombasthenia. However, routine blood tests such as complete blood count, liver function tests, and INR/PTT will not detect this disorder (platelet count and morphology are normal). The most widely used critical test for Glanzmann's thrombasthenia involves platelet aggregation challenges in which various physiologic agonists are mixed with platelets to induce clumping. Decreased platelet aggregation will occur after mixing with arachidonic acid, collagen, ADP, and thrombin; however, aggregation* will* occur in the presence of the antibiotic ristocetin, and this finding is the definitive diagnosis of Glanzmann's thrombasthenia [3, 5]. Recently, other tests and automated processes are becoming available such as direct analysis for the presence of glycoprotein IIb/IIIa and evaluation of whole blood samples with the PFA-100 impedance analyzer [3, 5, 6].

In general, hemarthrosis associated with Glanzmann's thrombasthenia is apparently very rare despite the common symptoms noted above. George et al. reported that hemarthrosis occurred in only 5 of 177 patients (3%) [1]. Only one of these had knee involvement. Urakawa et al. described a case of knee hemarthrosis in an 8-year-old boy after a sports-related injury. In the workup, he was diagnosed with Glanzmann's thrombasthenia [7]. The patient had a history of prolonged bleeding time after minor injuries and prolonged purpura. There was no family history, and previous workups did not identify the cause of the problems as being Glanzmann's thrombasthenia. Several reports in the literature describe perioperative management of patients with known Glanzmann's thrombasthenia [8–10]. Although cases similar to ours have been presented in which there were postoperative hemarthrosis complications in patients with previously unknown hemophilia or low factor XIII activity [11], we are unaware of previously reported cases of hemarthrosis after knee arthroscopy in a physically active adult man who had no knowledge of having Glanzmann's thrombasthenia.

We note that, rarely, Glanzmann's thrombasthenia may present as an acquired autoimmune disorder of platelet function, with rapid onset of bleeding events characterized by prolonged bleeding times but with normal platelet count and normal platelet glycoprotein expression [12]. This is caused by an antibody with specificity for platelet glycoprotein IIb/IIIa. Our patient had no history of immune thrombocytopenia or related conditions and thus acquired Glanzmann's thrombasthenia was considered highly unlikely.

## 4. Conclusion

Although counterintuitive in a physically active adult, clinical suspicion should include previously undiagnosed rare bleeding disorders if there is unusual and unexplained postoperative bleeding after knee arthroscopy. Once that line of thought is established, the actual definitive diagnosis of Glanzmann's thrombasthenia in particular can be readily made with the appropriate laboratory test.
